# LDL-cholesterol in newborns and children with genetically verified familial hypercholesterolaemia: implications for cholesterol-based screening

**DOI:** 10.1093/eurheartj/ehaf815

**Published:** 2025-10-23

**Authors:** Martin Prøven Bogsrud, Tonje Talsnes Stava, Knut Erik Berge, Thea Bismo Strøm, Kjetil Retterstøl, Kirsten B Holven

**Affiliations:** Unit for Cardiac and Cardiovascular Genetics, Department of Medical Genetics, Oslo University Hospital, P.O. Box 4950 Nydalen, NO-0484 Oslo, Norway; Unit for Cardiac and Cardiovascular Genetics, Department of Medical Genetics, Oslo University Hospital, P.O. Box 4950 Nydalen, NO-0484 Oslo, Norway; Department of Nutrition, Faculty of Medicine, University of Oslo, Oslo, Norway; Unit for Cardiac and Cardiovascular Genetics, Department of Medical Genetics, Oslo University Hospital, P.O. Box 4950 Nydalen, NO-0484 Oslo, Norway; Unit for Cardiac and Cardiovascular Genetics, Department of Medical Genetics, Oslo University Hospital, P.O. Box 4950 Nydalen, NO-0484 Oslo, Norway; Department of Nutrition, Faculty of Medicine, University of Oslo, Oslo, Norway; Lipid Clinic, Oslo University Hospital, Oslo, Norway; Department of Nutrition, Faculty of Medicine, University of Oslo, Oslo, Norway; Norwegian National Network on Familial Hypercholesterolemia, Oslo University Hospital, Oslo, Norway

**Keywords:** Familial hypercholesterolaemia, Screening, Children, Newborn screening

## Abstract

**Background and Aims:**

Cholesterol screening in children, with subsequent genetic testing of top percentile, has been suggested as an efficient universal screening approach in familial hypercholesterolaemia (FH). The potential cholesterol-based screening efficacy was investigated in a national genetically based screening programme.

**Methods:**

Data were from the Norwegian national family cascade screening programme in FH children from 1998 to 2023. Cholesterol levels [umbilical cord in newborns (*n* = 113) and venous blood in children 1–12 years old (*n* = 1346)] in variant positive and variant negative children were compared.

**Results:**

LDL cholesterol (LDL-C) was higher in FH newborns vs non-FH newborns [1.22 (.48) vs .68 (.32) mmol/L, *P* < .001], but overlapped widely. Cut-off levels corresponding to the 95th and 85th percentile would only identify 55.7% and 75.4% of newborns with FH, respectively. Screening efficacy in newborns did not differ in subgroups: boys and girls, null and non-null variants, variant gene, and neither for total cholesterol nor for non-HDL cholesterol. In all other age groups (from 1 to 12 years), LDL-C discriminated highly between mutation FH and non-FH children. Cut-off levels corresponding to 95th and 85th percentile of LDL-C would identify 88.4% and 94.1% of 1–12-year-old children with FH, respectively.

**Conclusions:**

Previous studies investigating lipid or genetic screening approaches for FH have limitations of only performing genetic testing in children with high LDL-C levels. The present study is the first to show the true LDL-C overlap in children with FH vs non-FH by utilizing unique data from a national family cascade screening programme. Cholesterol-based screening approaches for FH only seem feasible from 1 year of age onward.

## Structured Graphical Abstract

**Structured Graphical Abstract:**
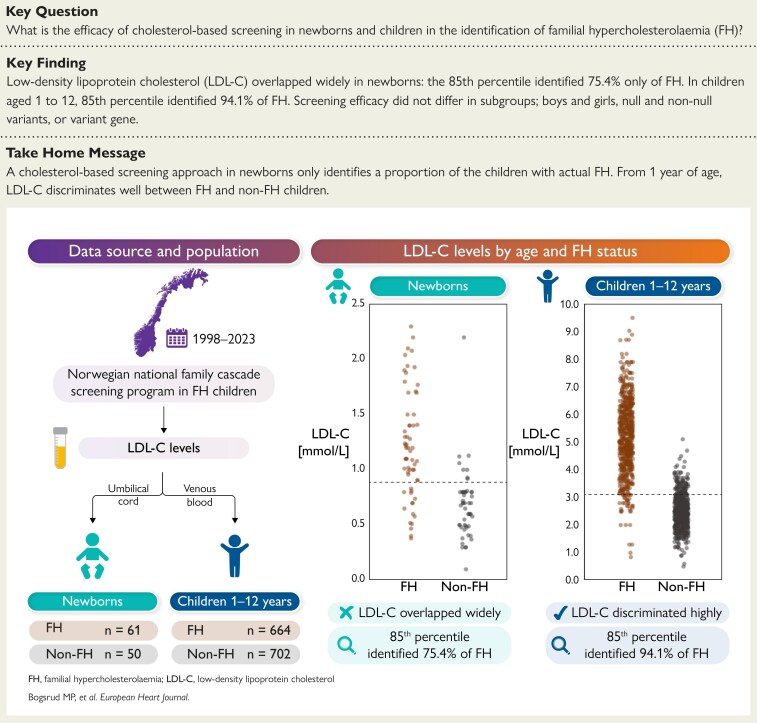
LDL cholesterol in newborns and children with genetically verified familial hypercholesterolaemia: implications for cholesterol-based screening. LDL-C, LDL cholesterol; y/o, years old; FH, familial hypercholesterolaemia.

**See the editorial comment for this article ‘Optimal timing for genetic verification of familial hypercholesterolaemia in children’, by S.T. Nielsen and R. Frikke-Schmidt, https://doi.org/10.1093/eurheartj/ehaf773.**

## Introduction

Familial hypercholesterolaemia (FH), caused by variants in the genes encoding LDL receptor (*LDLR*), apolipoprotein B (*APOB*), or proprotein convertase subtilisin/kexin Type 9 (*PCSK9*), is the most common autosomal dominant cause of premature cardiovascular disease (CVD).^1^ Early signs of atherosclerosis are evident in children before 8 years of age,^2^ and more than 90% experience CVD during adult life.^3^ Cholesterol-lowering treatment initiated in childhood is well documented and may eliminate the increased risk.^4,5^ Familial hypercholesterolaemia fulfils all Wilson and Jungner criteria for screening,^6^ and several initiatives are ongoing worldwide.^7–9^ Due to the cost and capacity of genetic testing, screening initiatives are suggested to be based on cholesterol measurements and subsequent genetic testing of the top percentile.^9^ However, although the hallmark of FH is generally hypercholesterolaemia, some children with FH present with normal cholesterol levels at one time point followed by an increase in cholesterol levels later in life and would thus be missed in lipid-screening approaches.^10^ Non-FH children may have hypercholesterolaemia due to polygenic or lifestyle factors.^11,12^ Further, due to the low FH prevalence of 1/300, analytical variations in lipids in non-FH children could potentially outnumber FH children in the top percentiles of lipid screening. Few and sparse data exist on the true overlap in lipid levels in children with and without genetically verified FH,^13,14^ and in order to evaluate the true effect of lipid-approached screening programmes, there is a need for more robust data. In this study, we have investigated cholesterol levels measured in umbilical cord blood from newborns and venous blood samples in children from 1 to 12 years old in the Norwegian national family cascade screening programme for families with FH.

## Methods

### Patients and study design

The study cohort comprise children participating in the Norwegian national family cascade screening programme for FH at the Unit for Cardiac and Cardiovascular Genetics from 1998 to 2023. More than 11 000 persons have been genetically diagnosed with FH in Norway, corresponding to more than 60% diagnosed of an estimated prevalence of 1/300. All genetic testing for FH in Norway has been performed by the Unit for Cardiac and Cardiovascular Genetics since 1998. Any doctor (e.g. family doctor or cardiologist) can free-of-charge request genetic testing in persons with high cholesterol, thus non-systematically opportunistic testing. If a pathogenic variant is detected, subsequent nationally organized systematically family screening of children (and other relatives) is performed, regardless of cholesterol measurements. Consequently, the children included in the study comprised an unselected (thus not based on pre-genetic-testing cholesterol measurements) population of children in which a pathogenic FH variant had been detected in one of the parents. Also, only families with definite pathogenic variants (according to the classification criteria by the American College of Medical Genetics and Genomics and the Association for Molecular Pathology and The Clinical Genome Resource Familial Hypercholesterolemia Variant Curation Expert Panel consensus guidelines for LDLR variant classification) in *LDLR*, *APOB*, and *PCSK9* were included (see Supplementary data online, *Table S1*  in the Supplementary Appendix).^15,16^ Genetic testing was limited to presence (or not) of the known pathogenic variant in the family. Data on age, sex, variant status, and untreated cholesterol levels were collected. Blood samples from newborns had been drawn from the umbilical cord. Non-newborn children were grouped according to the age when the venous blood sample was drawn (year sample draw—year of birth). For example, children in the 1-year group comprised children in which the venous blood sample was drawn at age 1–2 years (in the period from the first birthday to the day before the second birthday), the 2-year group comprised children in which the venous blood sample was drawn at age 2–3 years, and so on. Children in the 0-year group (*n* = 4), where a non-umbilical cord blood sample was taken after birth but before 1 year of age, were excluded from the analysis due to low sample size. Children who did not have a direct LDL cholesterol (LDL-C) measurement and for whom it was not possible to calculate LDL-C due to missing total cholesterol, HDL cholesterol (HDL-C), or triglycerides (*n* = 7) were also excluded. LDL-C levels were compared between variant positive and variant negative children, in total, within each age group and according to sex, gene, and genetic variant type (null vs non-null). Null variants were defined as all nonsense variants, frameshift variants, large deletions, large insertions, and splice site variants. Potential cholesterol screening efficacy was calculated for the 99th, 95th, 90th, 85th, and 80th percentile of LDL-C, based on LDL-C levels in variant negative children in each age cohort, thus corresponding to the normal population.

### Cholesterol measurements

All lipid values were measured (only once per child) at the Department for Medical Biochemistry, Oslo University Hospital, by the current standardized measurement method, mostly by direct measurement. LDL-C was calculated by using the Friedewald or Martin–Hopkins formula.^17,18^

### Statistics

To describe the distribution of LDL-C within the different subgroups, percentiles were calculated on the whole dataset including minimum and maximum values and possible outliers based on a linear interpolation method. In the absence of a sufficiently large independent dataset, cut-off values and percentiles for the different age groups and methods were found from the cohort without a pathogenic variant. The percentile groups included all in the given subgroups with LDL-C > the given percentile. The Shapiro–Wilk tests were performed to assess normality for groups with small sample sizes (*n* < 30). For groups with larger sample sizes (*n* ≥ 30), normality was assumed based on the central limit theorem. Because of possible significant variance between the groups, a Welch’s *t*-test was performed to compare the variant positive and variant negative individuals in the different groups. Comparisons of LDL-C by direct measurement and calculations were performed by independent *t*-test. Statistical analyses were performed using Stata version 16.1 (StataCorp. 2019. Stata Statistical Software: Release 16/. College Station, TX: StataCorp LLC, TX, USA). Data are presented as mean and standard deviation (SD) unless otherwise noted.

### Permission and ethics

Children participate in the Norwegian national family cascade screening programme following genetic counselling and written informed consent. The present data collection was performed by exporting fully anonymized data from the laboratory information system at the Unit for Cardiac and Cardiovascular Genetics. The study was evaluated by the Regional Research Ethics Committee (ref. no. 683460) to not require further consent from the patients. This study was funded by a research grant from the South-Eastern Norway Regional Health Authority (project no. 2020058).

## Results

### Patients

Of the 1459 children, 113 were newborns and 1346 were aged 1–12 years. Age at genetic testing was 7.2 (3.5) years and 52.5% were boys. All families harboured a definite pathogenic variant, and 706 (48.4%) of the children tested positive (heterozygous) for the variant in the family (see Supplementary data online, *Table S1*  in the Supplementary Appendix). Seven (1.0%) children harboured a mutation in *PCSK9*, 29 (4.1%) in *APOB*, and 670 (94.9%) in *LDLR*. Of the variants in *LDLR*, 272 (40.6%) were loss-of-function variants.

### Direct measured or calculated LDL cholesterol

Both direct measured and calculated LDL-C were available in 109 newborns and 1236 children aged 1–12 years. Calculated LDL-C in newborns was significantly higher than direct measured LDL-C for both the Friedewald and Martin–Hopkins equations in both FH and non-FH (*Table 1*). In children aged 1–12 years at genetic testing, calculated LDL-C by the Friedewald equation was not significantly different from direct measured LDL-C.

**Table 1:** Direct measured and calculated LDL cholesterol

	Direct measurement		Friedewald equation			Martin–Hopkins equation		
	*n*	LDL-C (mmol/L)	*n*	LDL-C (mmol/L)	*P*	*n*	LDL-C (mmol/L)	*P*
FH								
Newborns	59	1.21 (.49)	59	1.40 (.49)	.036	59	1.49 (.51)	.003
1–12 years old	588	5.08 (1.30)	588	5.19 (1.37)	.169	588	5.33 (1.37)	.002
Non-FH								
Newborns	50	.68 (.32)	50	.88 (.33)	.002	50	.96 (.35)	<.001
1–12 years old	648	2.47 (.59)	648	2.43 (.66)	.238	648	2.56 (.60)	.010

FH, familial hypercholesterolaemia; LDL-C, LDL cholesterol.

Data are presented as mean and standard deviation (SD).

An independent *t*-test was conducted to compare direct measurement vs Friedewald and Martin–Hopkins.

### Newborns

**Figure 1:**
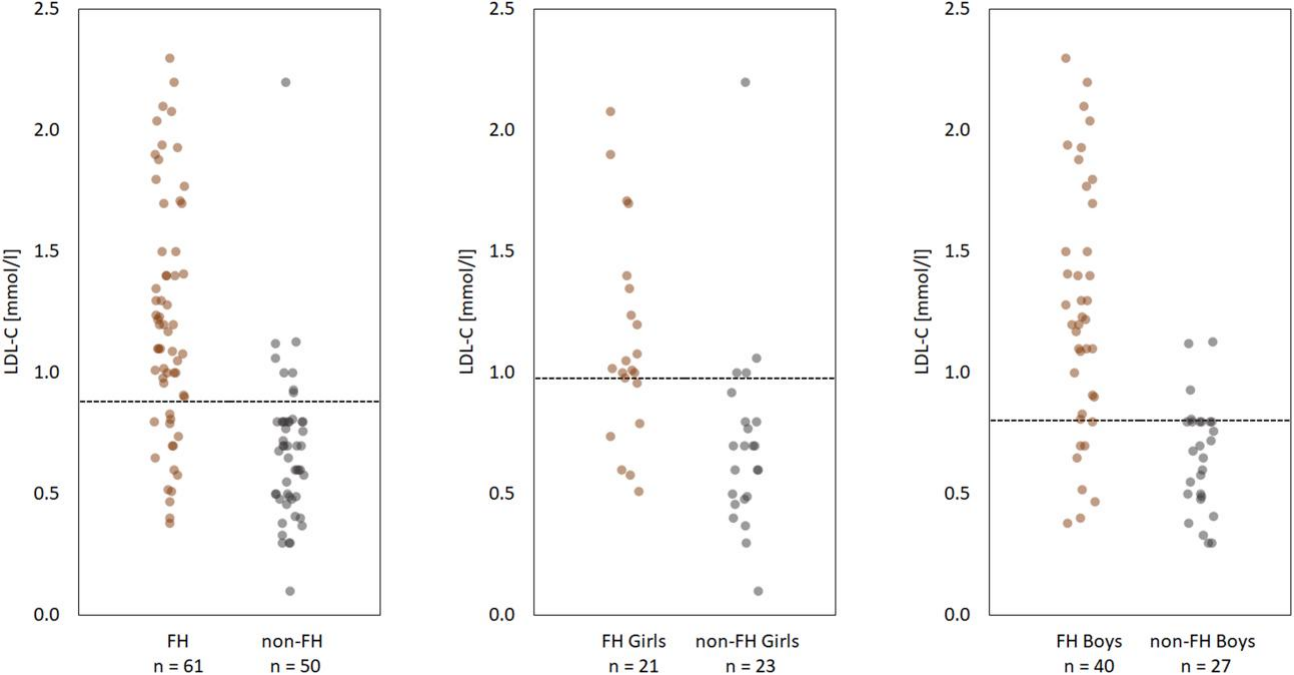
Direct measured LDL cholesterol in familial hypercholesterolaemia and non-familial hypercholesterolaemia newborns, in total and by sex. Dotted line indicates the 85th percentile cut-off

Total cholesterol levels were significantly higher in FH compared with non-FH newborns, but still low in both groups [2.45 (.62) vs 1.93 (.52) mmol/L; *P* < .001) (*Table 2*). Direct measured LDL-C was also low in both groups, but significantly higher in FH compared with non-FH newborns [1.22 (.48) vs .68 (.32) mmol/L; *P* < .001]. There was no difference between the groups in HDL-C [.81 (.25) vs .82 (.22) mmol/L] or triglycerides [.50 (.32) vs .46 (.21) mmol/L]. Although highly significant difference in mean direct measured LDL-C, the overlap between FH and non-FH newborns was substantial (*Figure 1* and *Table 3*). With cut-off value at the 95th and 85th percentile, this would only identify 55.7% and 75.4% of the newborns with FH, respectively. There was no difference in LDL-C in FH newborn boys vs FH newborn girls [1.26 (.08) vs 1.14 (.09) mmol/L; *P* = .372] (*Figure 1*). There was no difference in LDL-C in FH newborns with null vs non-null variant [1.26 (.50) vs 1.18 (.47) mmol/L; *P* = .552], and the overlap in screening efficacy was similar for all subgroups; null and non-null variants, variant gene, total cholesterol, non-HDL-C, and direct measured or calculated LDL-C (see Supplementary data online, *Figures S1*–*S3*  in the Supplementary Appendix).

**Table 2:** Cholesterol levels in familial hypercholesterolaemia and non-familial hypercholesterolaemia newborns

	FH		Non-FH		
	*n*	Mean (SD)	*n*	Mean (SD)	*P*
Total cholesterol	61	2.45 (.62)	51	1.93 (.52)	<.001
HDL cholesterol	61	.81 (.25)	51	.82 (.22)	.884
Triglycerides	61	.50 (.32)	51	.46 (.21)	.375
LDL cholesterol					
Direct measurement	61	1.22 (.48)	50	.68 (.32)	<.001
Martin–Hopkins equation	60	1.49 (.50)	51	.98 (.37)	<.001
Friedewald equation	60	1.41 (.49)	51	.90 (.35)	<.001
Non-HDL cholesterol	60	1.63 (.54)	51	1.11 (.41)	<.001

FH, familial hypercholesterolaemia.

Unit for cholesterol and triglyceride values is mmol/L.

An independent *t*-test was conducted to compare cholesterol levels in FH vs non-FH.

**Table 3:** Screening efficacy according to cholesterol levels and percentile in newborns

			Percentiles														
			80th			85th			90th			95th			99th		
Method	FH (*n*)	Non-FH (*n*)	LDL-C	FH	Non-FH	LDL-C	FH	Non-FH	LDL-C	FH	Non-FH	LDL-C	FH	Non-FH	LDL-C	FH	Non-FH
Total cholesterol	61	51	2.20	68.9%	11.8%	2.20	68.9%	11.8%	2.40	41.0%	9.8%	2.95	23.0%	5.9%	3.60	3.3%	2.0%
				(*n* = 42)	(*n* = 6)		(*n* = 42)	(*n* = 6)		(*n* = 25)	(*n* = 5)		(*n* = 14)	(*n* = 3)		(*n* = 2)	(*n* = 1)
LDL-C																	
Direct measurement	61	50	.80	78.7%	18.0%	.88	75.4%	16.0%	1.00	63.9%	8.0%	1.09	55.7%	6.0%	1.68	23.0%	2.0%
				(*n* = 48)	(*n* = 9)		(*n* = 46)	(*n* = 8)		(*n* = 39)	(*n* = 4)		(*n* = 34)	(*n* = 3)		(*n* = 14)	(*n* = 1)
Martin–Hopkins	60	51	1.17	71.7%	19.6%	1.27	68.3%	15.7%	1.30	68.3%	9.8%	1.67	30.0%	5.9%	2.13	13.3%	2.0%
				(*n* = 43)	(*n* = 10)		(*n* = 41)	(*n* = 8)		(*n* = 41)	(*n* = 5)		(*n* = 18)	(*n* = 3)		(*n* = 8)	(*n* = 1)
Friedewald	60	51	1.06	73.3%	19.6%	1.22	65.0%	13.7%	1.24	65.0%	9.8%	1.54	33.3%	5.9%	1.97	16.7%	2.0%
				(*n* = 44)	(*n* = 10)		(*n* = 39)	(*n* = 7)		(*n* = 39)	(*n* = 5)		(*n* = 20)	(*n* = 3)		(*n* = 10)	(*n* = 1)
Non-HDL	60	51	1.32	68.3%	19.6%	1.40	66.7%	9.8%	1.40	66.7%	9.8%	1.90	26.7%	5.9%	2.40	8.3%	0%
				(*n* = 41)	(*n* = 10)		(*n* = 40)	(*n* = 5)		(*n* = 40)	(*n* = 5)		(*n* = 16)	(*n* = 3)		(*n* = 5)	(*n* = 0)

FH, familial hypercholesterolaemia; LDL-C; LDL cholesterol.

Percentiles were calculated using an inclusive percentile calculation method including minimum and maximum values based on a linear interpolation method and the specific percentiles includes all with LDL-C > the given percentile.

The combined group includes direct measurements of LDL cholesterol, supplemented by LDL levels calculated using the Friedewald equation when direct measurements were unavailable.

### One to twelve year-old children

**Figure 2:**
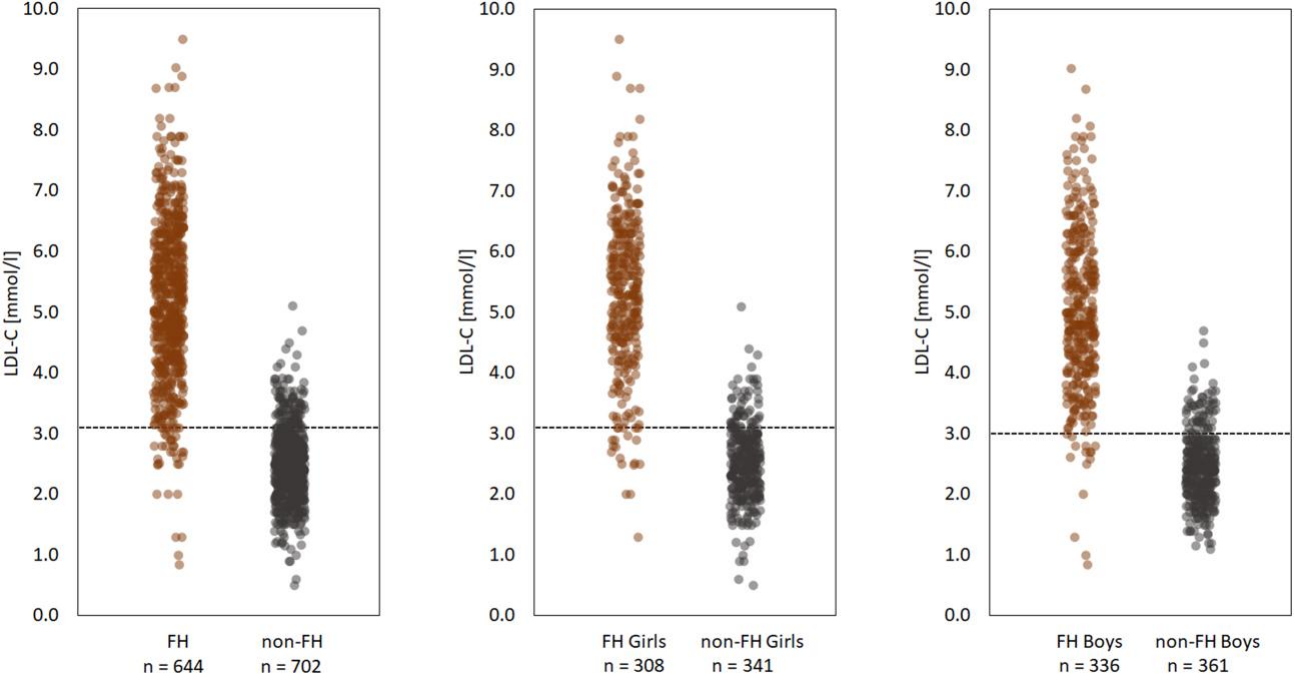
LDL cholesterol in familial hypercholesterolaemia and non-familial hypercholesterolaemia children aged 1–12 years, in total and by sex. Dotted line indicates the 85th percentile cut-off

LDL-C was significantly higher in variant positive (*n* = 644) compared with variant negative (*n* = 702) children aged 1–12 years at genetic testing [5.16 (1.31) mmol/L vs 2.49 (.60); *P* < .001, respectively] (*Table 4*). LDL-C discriminated well between FH and non-FH children; the 95th and 85th percentile would identify 88.4% and 94.1% of FH, respectively (*Figure 2* and *Table 5*). LDL-C was higher in 1–12-year-old FH girls vs boys [5.32 (1.30) vs 5.01 (1.31) mmol/L; *P* = .003]; the 95th and 85th percentile would identify 89.9% and 93.5% of girls, respectively, and 87.8% and 95.5% boys, respectively (*Figure 2*). LDL-C was higher in 1–12-year-old FH children with null vs non-null variants [5.71 (1.18) vs 4.82 (1.28); *P* < .001); the 95th and 85th percentile would identify 95.5% and 98.4% of null variants and 86.0% and 93.5% of non-null variants (see Supplementary data online, *Figure S4*  in the Supplementary Appendix). The overlap was similar according to variant gene and in each individual age group (see Supplementary data online, *Figures S5* and *S6*  in the Supplementary Appendix).

**Table 4:** LDL cholesterol in familial hypercholesterolaemia and non-familial hypercholesterolaemia children aged 1–12 years

		FH		Non-FH	
Age (years)	*n*	LDL-C (mmol/L)	*n*	LDL-C (mmol/L)	*P*
1	14	5.23 (1.76)	11	1.93 (.32)	<.001
2	20	4.61 (1.36)	21	2.49 (.68)	<.001
3	36	5.56 (1.10)	31	2.37 (.66)	<.001
4	35	5.45 (1.41)	46	2.48 (.65)	<.001
5	57	5.22 (1.28)	54	2.52 (.52)	<.001
6	49	5.16 (1.47)	72	2.50 (.67)	<.001
7	67	5.12 (1.26)	62	2.55 (.57)	<.001
8	71	4.85 (1.34)	80	2.52 (.59)	<.001
9	77	5.41 (1.22)	64	2.58 (.62)	<.001
10	84	5.00 (1.14)	85	2.51 (.61)	<.001
11	75	5.32 (1.42)	93	2.52 (.62)	<.001
12	59	4.92 (1.27)	83	2.43 (.52)	<.001
1–12	644	5.16 (1.31)	702	2.49 (.60)	<.001

FH, Familial hypercholesterolaemia; LDL-C, LDL cholesterol.

Data are presented as mean and standard deviation (SD) unless otherwise noted.

Direct measured LDL cholesterol was used if available, otherwise calculated LDL cholesterol by Friedewald was used (*n* = 109).

**Table 5:** Screening efficacy according to LDL cholesterol and percentiles in children aged 1–12 years

			80th			85th			90th			95th			99th		
Age	FH	Non-FH	LDL-C	FH	Non-FH	LDL-C	FH	Non-FH	LDL-C	FH	Non-FH	LDL-C	FH	Non-FH	LDL-C	FH	Non-FH
(years)	(*n*)	(*n*)	(mmol/L)			(mmol/L)			(mmol/L)			(mmol/L)			(mmol/L)		
1	14	11	2.20	92.9%	18.2%	2.22	92.9%	18.2%	2.24	92.9%	9.1%	2.29	92.9%	9.1%	2.32	92.9%	9.1%
				(*n* = 13)	(*n* = 2)		(*n* = 13)	(*n* = 2)		(*n* = 13)	(*n* = 1)		(*n* = 13)	(*n* = 1)		(*n* = 13)	(*n* = 1)
2	20	21	3.00	85.0%	19.0%	3.13	85.0%	14.3%	3.20	85.0%	9.5%	3.30	80.0%	4.8%	3.54	70.0%	4.8%
				(*n* = 17)	(*n* = 4)		(*n* = 17)	(*n* = 3)		(*n* = 17)	(*n* = 2)		(*n* = 16)	(*n* = 1)		(*n* = 14)	(*n* = 1)
3	36	31	3.00	100.0%	16.1%	3.05	100.0%	16.1%	3.16	100.0%	9.7%	3.53	94.4%	6.4%	3.69	94.4%	3.2%
				(*n* = 36)	(*n* = 5)		(*n* = 36)	(*n* = 5)		(*n* = 36)	(*n* = 3)		(*n* = 34)	(*n* = 2)		(*n* = 34)	(*n* = 1)
4	35	46	3.00	94.3%	19.6%	3.34	91.4%	15.2%	3.49	91.4%	10.9%	3.58	88.6%	6.5%	3.74	88.6%	2.2%
				(*n* = 33)	(*n* = 9)		(*n* = 32)	(*n* = 7)		(*n* = 32)	(*n* = 5)		(*n* = 31)	(*n* = 3)		(*n* = 31)	(*n* = 1)
5	57	54	2.90	94.7%	14.8%	2.91	94.7%	14.8%	3.10	93.0%	11.1%	3.42	93.0%	5.6%	3.78	93.0%	1.9%
				(*n* = 54)	(*n* = 8)		(*n* = 54)	(*n* = 8)		(*n* = 53)	(*n* = 6)		(*n* = 53)	(*n* = 3)		(*n* = 53)	(*n* = 1)
6	49	72	3.00	95.9%	18.1%	3.30	85.7%	12.5%	3.39	85.7%	11.1%	3.53	83.7%	5.6%	3.90	79.6%	1.4%
				(*n* = 47)	(*n* = 13)		(*n* = 42)	(*n* = 9)		(*n* = 42)	(*n* = 8)		(*n* = 41)	(*n* = 4)		(*n* = 39)	(*n* = 1)
7	67	62	2.91	97.0%	19.4%	2.99	97.0%	16.1%	3.19	95.5%	11.3%	3.70	88.1%	4.8%	4.06	77.6%	1.6%
				(*n* = 65)	(*n* = 12)		(*n* = 65)	(*n* = 10)		(*n* = 64)	(*n* = 7)		(*n* = 59)	(*n* = 3)		(*n* = 52)	(*n* = 1)
8	71	80	3.09	91.5%	20.0%	3.13	91.5%	15.0%	3.22	90.1%	10.0%	3.41	87.3%	5.0%	3.71	83.1%	1.3%
				(*n* = 65)	(*n* = 16)		(*n* = 65)	(*n* = 12)		(*n* = 64)	(*n* = 8)		(*n* = 62)	(*n* = 4)		(*n* = 59)	(*n* = 1)
9	77	64	2.96	100.0%	20.3%	3.11	97.4%	15.6%	3.31	96.1%	10.9%	3.85	90.9%	6.3%	4.44	79.2%	1.6%
				(*n* = 77)	(*n* = 13)		(*n* = 75)	(*n* = 10)		(*n* = 74)	(*n* = 7)		(*n* = 70)	(*n* = 4)		(*n* = 61)	(*n* = 1)
10	84	85	2.81	98.8%	20.0%	2.94	98.8%	15.3%	3.10	96.4%	9.4%	3.51	88.1%	5.9%	4.76	54.8%	1.2%
				(*n* = 83)	(*n* = 17)		(*n* = 83)	(*n* = 13)		(*n* = 81)	(*n* = 8)		(*n* = 74)	(*n* = 5)		(*n* = 46)	(*n* = 1)
11	75	93	2.91	94.7%	19.4%	3.11	93.3%	15.1%	3.29	89.3%	10.8%	3.70	82.7%	4.3%	4.10	81.3%	1.1%
				(*n* = 71)	(*n* = 18)		(*n* = 70)	(*n* = 14)		(*n* = 67)	(*n* = 10)		(*n* = 62)	(*n* = 4)		(*n* = 61)	(*n* = 1)
12	59	83	2.86	94.9%	19.3%	2.97	91.5%	15.7%	3.10	91.5%	9.6%	3.25	89.8%	4.8%	3.50	86.4%	1.2%
				(*n* = 56)	(*n* = 16)		(*n* = 54)	(*n* = 13)		(*n* = 54)	(*n* = 8)		(*n* = 53)	(*n* = 4)		(*n* = 51)	(*n* = 1)
1–12	644	702	2.91	95.8%	19.9%	3.10	94.1%	13.5%	3.30	91.3%	9.3%	3.56	88.4%	5.0%	4.10	79.3%	.9%
				(*n* = 617)	(*n* = 140)		(*n* = 606)	(*n* = 95)		(*n* = 588)	(*n* = 65)		(*n* = 570)	(*n* = 35)		(*n* = 511)	(*n* = 6)

LDL-C, LDL cholesterol.

Percentiles calculated in the combined 1–12-year group are naturally slightly different from percentiles calculated in the individual groups; e.g. LDL of 2.91 mmol/L represents the 80th percentile in the combined group of all children aged 1–12 years, while an LDL of 3.09 mmol/L represents the 80th percentile if calculated only in 8-year-old children. Thus, some 8-year-old children not exceeding the 80th percentile in the 8-year group will exceed the 80th percentile based on the combined group of children aged from 1 to 12 years. Thus, the sum of children exceeding the different percentiles in each individual age group will not exactly match the sum in total group.

## Discussion

The present study is the first to describe detailed large data on overlap in LDL-C between definite FH and definite non-FH children. These findings are essential in order to evaluate the effectiveness of cholesterol-based screening programmes.

Held *et al*.^19^ recently validated cholesterol measurements in dried blood spots (DBSs) collected for newborn screening. LDL-C levels in 10 004 newborns displayed a normal distribution with a skewed tail of newborns with hypercholesterolaemia, potentially being a small group of newborns with genetically raised LDL-C.^20^ However, pre-genetic-testing era cholesterol-based screening efforts to detect FH indicated that umbilical cord blood cholesterol could not predict hypercholesterolaemia neither in the parents nor persistent high cholesterol at recurrent measurement in the child.^21^ Newborn lipid levels in genetic FH have previously only been investigated in one small study by Vuorio *et al*.,^14^ showing low LDL-C and substantial overlap between 11 FH newborns and 14 non-FH newborn controls. These findings are confirmed and further quantified in the present larger study of 61 FH newborns and 50 non-FH newborn controls. In newborns, an LDL-C-based cut-off corresponding to the 95th and 85th percentile would only identify 55.7% and 75.4% of genetic FH, respectively. If possible to implement in existing newborn screening programmes at low cost, such diagnostic yield would be better than no screening. Considering a prevalence of FH of 1/300, 61 identified genetically verified FH newborns correspond to almost 20 000 newborns, so the present data comparing genetically FH with non-FH are a robust comparison. Newborn screening for FH is currently performed in the Czech Republic, by LDL-C measurement in umbilical cord blood and subsequent genetic testing if above the 85th percentile.^8^ Data from this newborn screening programme are not yet published, but based on the present data, a screening efficacy of ∼75% would be expected.

The hallmark of FH is increased LDL-C, normally contributing to 70% of the cholesterol content in blood. However, newborns generally display a lipid profile distinct from that of older children or adults, with HDL being the predominant lipid carrier.^22^ This is also evident from the present study. The distinct lipid profile in newborns is also evident by the fact that LDL-C calculations in the present study overestimated LDL-C by 29% in non-FH and 17% in FH, compared with direct measured LDL-C. In 1–12-year-old children, there were no differences in direct measured vs calculated LDL-C.

LDL-C levels are very low in newborns and overlapped widely in FH and non-FH. The low LDL-C could possibly indicate that the LDL precursor very LDL (VLDL) is less important in energy consumption in foetus, thus not yielding amounts of LDL-C dependent on the LDL receptor for clearance. At the very low newborn LDL-C levels, regardless of cause, the hallmark of FH, fewer functional LDL receptors, might not play as important role as later in life when LDL-C is higher. Thus, the wider overlap between FH and non-FH newborns, compared with all other age groups, could be explained by the less importance of LDL receptors in foetal and newborn metabolism.

From 1 year of age, LDL-C increase to normal levels in controls, and FH exhibits the typical doubling of normal LDL-C, as also shown by Vuorio *et al*. in a small number of patients.^14,23^ Wald *et al*.^13^ investigated the overlap in cholesterol levels between FH and non-FH in capillary blood samples from 10 065 children, collected at vaccination visits at age 1–2 years. Total cholesterol measurements and genetic testing for 48 known FH variants were performed in all children. Thirty pathogenic FH variants were detected, 13 of which were found in children with total cholesterol above the 99th percentile. Seventy-nine children with total cholesterol above the 99th percentile and no detected pathogenic variants underwent extended genetic testing including DNA sequencing of all exons in *LDLR*, Exon 7 in *PCSK9*, Exon 26 in *APOB*, and del/dup analysis of *LDLR*. The extended genetic testing identified seven pathogenic variants. No extended genetic testing was performed in children with cholesterol below the 99th percentile. The study by Wald exemplifies how a cholesterol-based screening approach, without gold standard genetic testing in all children (also those with lower cholesterol levels), cannot truly predict screening efficacy. In the present study, children were tested for a known variant in the family. Thus, the likelihood of undetected FH variant in hypercholesterolaemic non-FH newborns in the present study is negligible.

A cholesterol-based screening approach would not detect children with a pathogenic FH variant, but only moderately elevated cholesterol levels. Such approaches have been advocated by Wald *et al*., suggesting that harbouring a pathogenic FH variant with normal cholesterol levels is not a true FH. We previously showed that as many as 70% of children carrying an FH-causing variant, despite relatively normal LDL-C at the time of diagnosis, later require cholesterol-lowering treatment during a 10-year follow-up.^10^ Thus, low LDL-C at diagnosis in children does not exclude a pathogenic FH variant.

An FH screening programme should aim not only to diagnose but to have a clinical implication by starting a preventive treatment in time. Statin treatment is currently recommended for children with FH starting at 8–10 years of age. If the diagnosis is made at birth, who is responsible for ensuring the statin treatment begins 8 years later? The present data did not include information on the age at which statin treatment was started in relation to the age at diagnosis. However, a previous smaller study on FH children in Norway found that the average age for starting statin treatment was 12.5 years, despite the diagnosis being made years earlier. Thus, the challenge in preventing early disease in FH is not only diagnosis but also follow-up.^24^

The strength of the present study includes the unselected inclusion of a national screening programme. The control group comprises children who tested negative for the known familial mutation; although this offers a clean comparator group for internal validity, these children may not fully represent LDL-C distributions in the general paediatric population where polygenic hypercholesterolaemia and environmental factors may be more prevalent. Thus, a limitation of the study is a possible slight overestimation of screening efficacy.

In conclusion, there is a substantial overlap in LDL-C levels in FH compared with non-FH newborns. A cholesterol-based screening approach in newborns would only identify a proportion of the children with actual FH. From 1 year of age, LDL-C discriminates well between FH and non-FH children.

## Supplementary Material

ehaf815_Supplementary_Data

## Declarations

### Data Availability

All data available upon reasonable request.
