# Supplementary material for: LDL-cholesterol in newborns and children with genetically verified familial hypercholesterolaemia: implications for cholesterol-based screening
Source: Eur Heart J. 2025 Oct 23;46(48):5261–9. doi: 10.1093/eurheartj/ehaf815 (PMC12718687; doi:10.1093/eurheartj/ehaf815)

Table S1. Definite pathogenic variants in 706 FH children.

Gene	Transcript	Variant (nucleotide)	Exon/intron	Variant (protein)*	Variant (protein)	Variant (type)	Number of children	Null variant
LDLR	NM_000527.5	c.-136C>T	Upstream (signal peptide)	p.-	p.-	-	2	Yes
LDLR	NM_000527.5	c.-142C>T	Upstream (signal peptide)	p.-	p.-	-	1	Yes
LDLR	NM_000527.5	c.44T>A	Exon 1	p.(Leu-7His)	p.(Leu15His)	Missense	3	No
LDLR	NM_000527.5	c.81C>G	Exon 2	p.(Cys6Trp)	p.(Cys27Trp)	Missense	4	No
LDLR	NM_000527.5	c.131G>A	Exon 2	p.(Trp23*)	p.(Trp44*)	Nonsense	23	Yes
LDLR	NM_000527.5	c.259T>G	Exon 3	p.(Trp66Gly)	p.(Trp87Gly)	Missense	25	No
LDLR	NM_000527.5	c.270T>G	Exon 3	p.(Asp69Glu)	p.(Asp90Glu)	Missense	1	No
LDLR	NM_000527.5	c.296C>G	Exon 3	p.(Ser78*)	p.(Ser99*)	Nonsense	34	Yes
LDLR	NM_000527.5	c.313+1_313+2dup	Intron 3	p.-	p.-	Essential splice site	1	Yes
LDLR	NM_000527.5	c.313+1G>A	Intron 3	p.-	p.-	Essential splice site	120	Yes
LDLR	NM_000527.5	c.325T>C	Exon 4	p.(Cys88Arg)	p.(Cys109Arg)	Missense	3	No
LDLR	NM_000527.5	c.376T>C	Exon 4	p.(Phe105Leu)	p.(Phe126Leu)	Missense	1	No
LDLR	NM_000527.5	c.409G>A	Exon 4	p.(Gly116Ser)	p.(Gly137Ser)	Missense	1	No
LDLR	NM_000527.5	c.429C>A	Exon 4	p.(Cys122*)	p.(Cys143*)	Nonsense	2	Yes
LDLR	NM_000527.5	c.465C>A	Exon 4	p.(Cys134*)	p.(Cys155*)	Nonsense	12	Yes
LDLR	NM_000527.5	c.488dup	Exon 4	p.(Leu143Alafs*16)	p.(Leu164Alafs*16)	Frameshift	4	Yes
LDLR	NM_000527.5	c.502G>T	Exon 4	p.(Asp147Tyr)	p.(Asp168Tyr)	Missense	1	No
LDLR	NM_000527.5	c.523G>T	Exon 4	p.(Asp154Tyr)	p.(Asp175Tyr)	Missense	1	No
LDLR	NM_000527.5	c.536A>G	Exon 4	p.(Glu158Gly)	p.(Glu179Gly)	Missense	1	No
LDLR	NM_000527.5	c.563_569del	Exon 4	p.(Tyr167Serfs*16)	p.(Tyr188Serfs*16)	Frameshift	1	Yes
LDLR	NM_000527.5	c.647G>A	Exon 4	p.(Cys195Tyr)	p.(Cys216Tyr)	Missense	2	No
LDLR	NM_000527.5	c.661G>A	Exon 4	p.(Asp200Asn)	p.(Asp221Asn)	Missense	42	No
LDLR	NM_000527.5	c.662A>G	Exon 4	p.(Asp200Gly)	p.(Asp221Gly)	Missense	5	No
LDLR	NM_000527.5	c.664_681dup	Exon 4	p.(Cys201_Asp206dup)	p.(Cys222_Asp227dup)	In-frame	2	Yes
LDLR	NM_000527.5	c.682G>T	Exon 4	p.(Glu207*)	p.(Glu228*)	Nonsense	6	Yes

LDLR	NM_000527.5	c.691T>G	Exon 4	p.(Cys210Gly)	p.(Cys231Gly)	Missense	85	No
LDLR	NM_000527.5	c.708dup	Exon 5	p.(Arg216Serfs*3)	p.(Arg237Serfs*3)	Frameshift	1	Yes
LDLR	NM_000527.5	c.796G>A	Exon 5	p.(Asp245Asn)	p.(Asp266Asn)	Missense	5	No
LDLR	NM_000527.5	c.798T>A	Exon 5	p.(Asp245Glu)	p.(Asp266Glu)	Missense	3	No
LDLR	NM_000527.5	c.850T>A	Exon 6	p.(Cys263Ser)	p.(Cys284Ser)	Missense	2	No
LDLR	NM_000527.5	c.858C>G	Exon 6	p.(Ser265Arg)	p.(Ser286Arg)	Missense	1	No
LDLR	NM_000527.5	c.937T>G	Exon 6	p.(Cys292Gly)	p.(Cys313Gly)	Missense	1	No
LDLR	NM_000527.5	c.940+2T>C	Intron 6	p.-	p.-	Essential splice site	1	Yes
LDLR	NM_000527.5	c.986G>T	Exon 7	p.(Cys308Phe)	p.(Cys329Phe)	Missense	1	No
LDLR	NM_000527.5	c.1020C>A	Exon 7	p.(Cys319*)	p.(Cys340*)	Nonsense	2	Yes
LDLR	NM_000527.5	c.1060+1G>T	Intron 7	p.-	p.-	Essential splice site	1	Yes
LDLR	NM_000527.5	c.1066G>T	Exon 8	p.(Asp335Tyr)	p.(Asp356Tyr)	Missense	4	No
LDLR	NM_000527.5	c.1069G>A	Exon 8	p.(Glu336Lys)	p.(Glu357Lys)	Missense	1	No
LDLR	NM_000527.5	c.1097A>G	Exon 8	p.(Gln345Arg)	p.(Gln366Arg)	Missense	12	No
LDLR	NM_000527.5	c.1108A>C	Exon 8	p.(Asn349His)	p.(Asn370His)	Missense	1	No
LDLR	NM_000527.5	c.1145G>T	Exon 8	p.(Gly361Val)	p.(Gly382Val)	Missense	2	No
LDLR	NM_000527.5	c.1150C>T	Exon 8	p.(Gln363*)	p.(Gln384*)	Nonsense	1	Yes
LDLR	NM_000527.5	c.1176C>A	Exon 8	p.(Cys371*)	p.(Cys392*)	Nonsense	2	Yes
LDLR	NM_000527.5	c.1186+5G>A	Intron 8	p.-	p.-	-	1	Yes
LDLR	NM_000527.5	c.1201C>G	Exon 9	p.(Leu380Val)	p.(Leu401Val)	Missense	25	No
LDLR	NM_000527.5	c.1246C>T	Exon 9	p.(Arg395Trp)	p.(Arg416Trp)	Missense	70	No
LDLR	NM_000527.5	c.1247G>A	Exon 9	p.(Arg395Gln)	p.(Arg416Gln)	Missense	1	No
LDLR	NM_000527.5	c.1324T>C	Exon 9	p.(Tyr421His)	p.(Tyr442His)	Missense	1	No
LDLR	NM_000527.5	c.1342C>T	Exon 9	p.(Gln427*)	p.(Gln448*)	Nonsense	1	Yes
LDLR	NM_000527.5	c.1358+2T>A	Intron 9	p.-	p.-	Essential splice site	1	Yes
LDLR	NM_000527.5	c.1444G>A	Exon 10	p.(Asp461Asn)	p.(Asp482Asn)	Missense	4	No
LDLR	NM_000527.5	c.1469G>A	Exon 10	p.(Trp469*)	p.(Trp490*)	Nonsense	4	Yes
LDLR	NM_000527.5	c.1478_1479del	Exon 10	p.(Ser472Cysfs*42)	p.(Ser493Cysfs*42)	Frameshift	1	Yes
LDLR	NM_000527.5	c.1618G>A	Exon 11	p.(Ala519Thr)	p.(Ala540Thr)	Missense	1	No
LDLR	NM_000527.5	c.1646G>A	Exon 11	p.(Gly528Asp)	p.(Gly549Asp)	Missense	1	No
LDLR	NM_000527.5	c.1686G>A	Exon 11	p.(Trp541*)	p.(Trp562*)	Nonsense	8	Yes

LDLR	NM_000527.5	c.1727A>G	Exon 12	p.(Tyr555Cys)	p.(Tyr576Cys)	Missense	15	No
LDLR	NM_000527.5	c.1846-1G>A	Intron 12	p.-	p.-	Essential splice site	1	Yes
LDLR	NM_000527.5	c.2054C>T	Exon 14	p.(Pro664Leu)	p.(Pro685Leu)	Missense	53	No
LDLR	NM_000527.5	c.2140+86C>G	Intron 14	p.-	p.-	-	2	Yes
LDLR	NM_000527.5	c.2230C>T	Exon 15	p.(Arg723*)	p.(Arg744*)	Nonsense	1	Yes
LDLR	NM_000527.5	c.2413G>A	Exon 17	p.(Gly784Arg)	p.(Gly805Arg)	Missense	4	No
LDLR	NM_000527.5	c.2416dup	Exon 17	p.(Val785Glyfs*11)	p.(Val806Glyfs*11)	Frameshift	2	Yes
LDLR	NM_000527.5	c.2475C>G	Exon 17	p.(Asn804Lys)	p.(Asn825Lys)	Missense	15	No
LDLR	NM_000527.5	c.2500del	Exon 17	p.(Asp813Metfs*95)	p.(Asp834Metfs*95)	Frameshift	1	Yes
LDLR	NM_000527.5	c.-	Exon 11_14del	p.-	p.-	-	5	Yes
LDLR	NM_000527.5	c.-	Exon 11_18del	p.-	p.-	-	12	Yes
LDLR	NM_000527.5	c.-	Exon 2_3del	p.-	p.-	-	4	Yes
LDLR	NM_000527.5	c.-	Exon 2del	p.-	p.-	-	2	Yes
LDLR	NM_000527.5	c.-	Exon 4_6del	p.-	p.-	-	1	Yes
LDLR	NM_000527.5	c.-	Exon 7_10dup	p.-	p.-	-	3	Yes
LDLR	NM_000527.5	c.-	Exon 9_10dup	p.-	p.-	-	6	Yes
APOB	NM_000384.3	c.10580G>A	Exon 26	p.(Arg3500Gln)	p.(Arg3527Gln)	Missense	29	No
PCSK9	NM_174936.4	c.644G>A	Exon 4	p.(Arg215His)	p.(Arg215His)	Missense	6	No
PCSK9	NM_174936.4	c.1120G>T	Exon 7	p.(Asp374Tyr)	p.(Asp374Tyr)	Missense	1	No

**Excluding the 21 amino acid signal peptide*

Figure S1. LDL-C in FH and non-FH newborns by null and non-null variants.

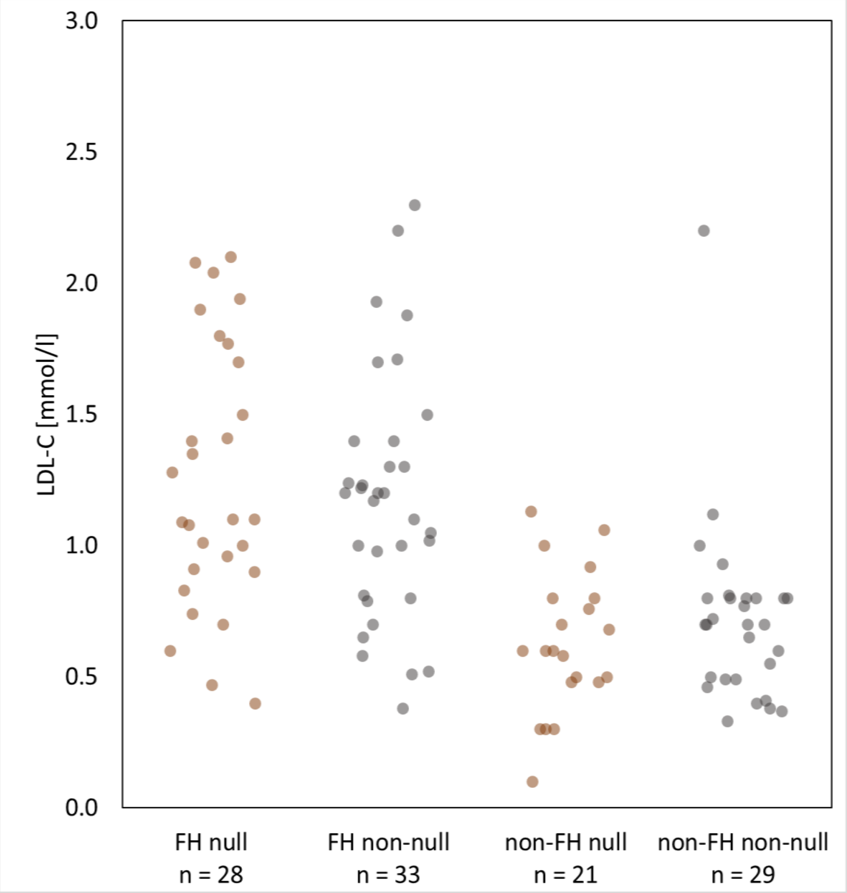

Figure S2. LDL-C in FH and non-FH newborns by variant gene.

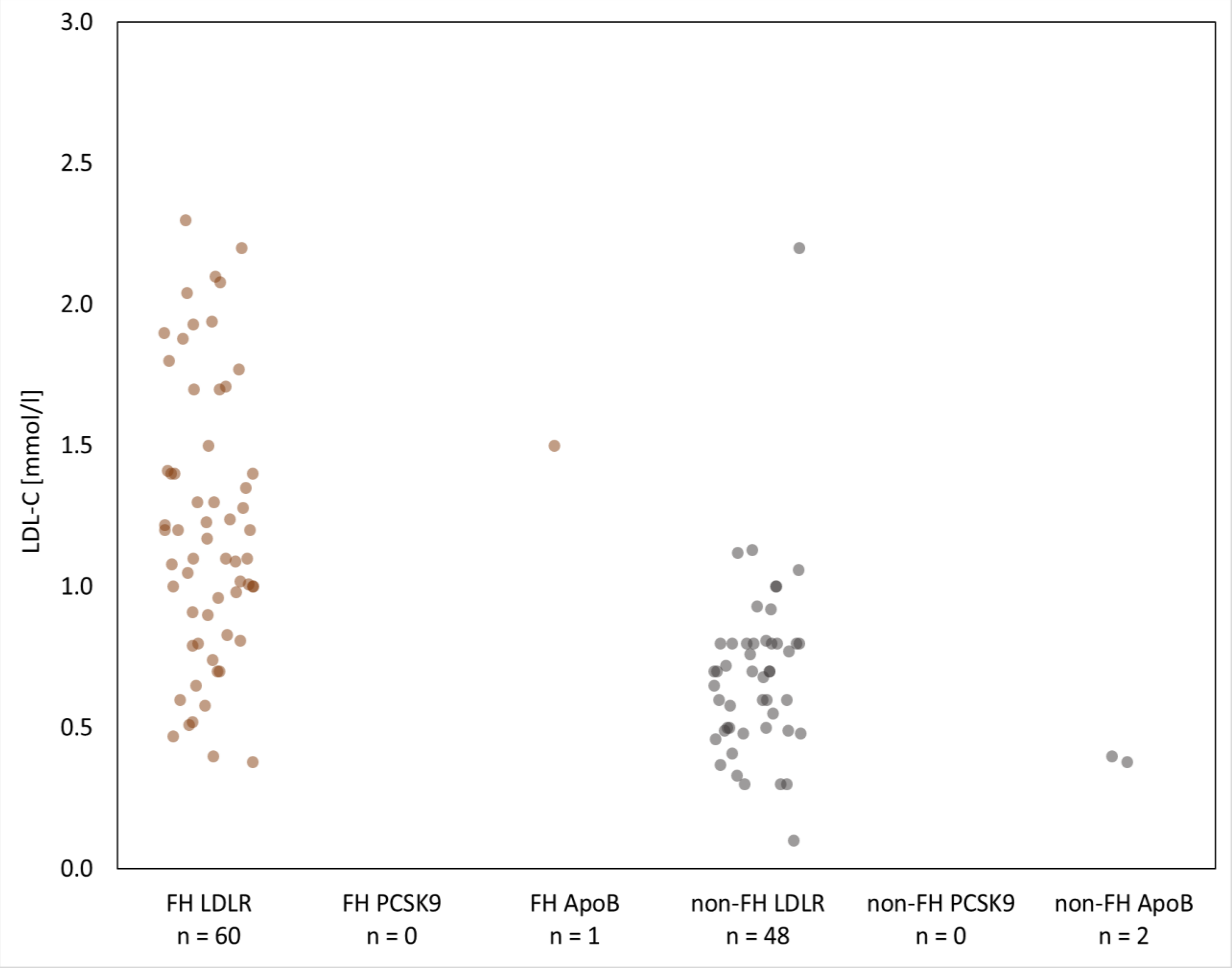

Figure S3. LDL-C in FH and non-FH newborns by cholesterol measurement method.

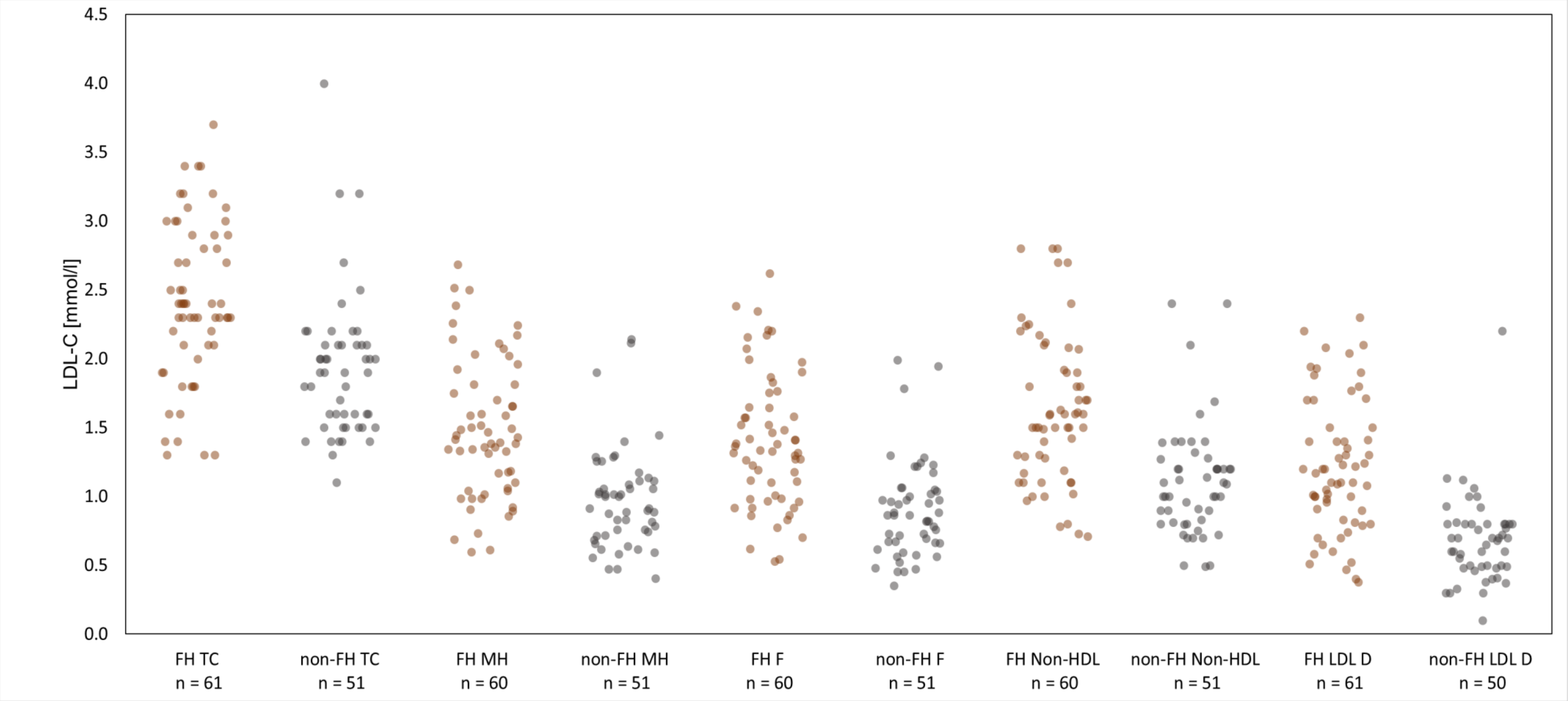

FH, Familial hypercholesterolemia; LDL-C, low density lipoprotein cholesterol; TC, measured total cholesterol; MH, Martin Hopkins equation; F, Friedewalds equation; Non-HDL, Measured total cholesterol-measured high density lipoprotein; LDL D, directly measured low density lipoprotein

Figure S4. LDL-C in FH and non-FH children 1-12 year old by null and non-null variants.

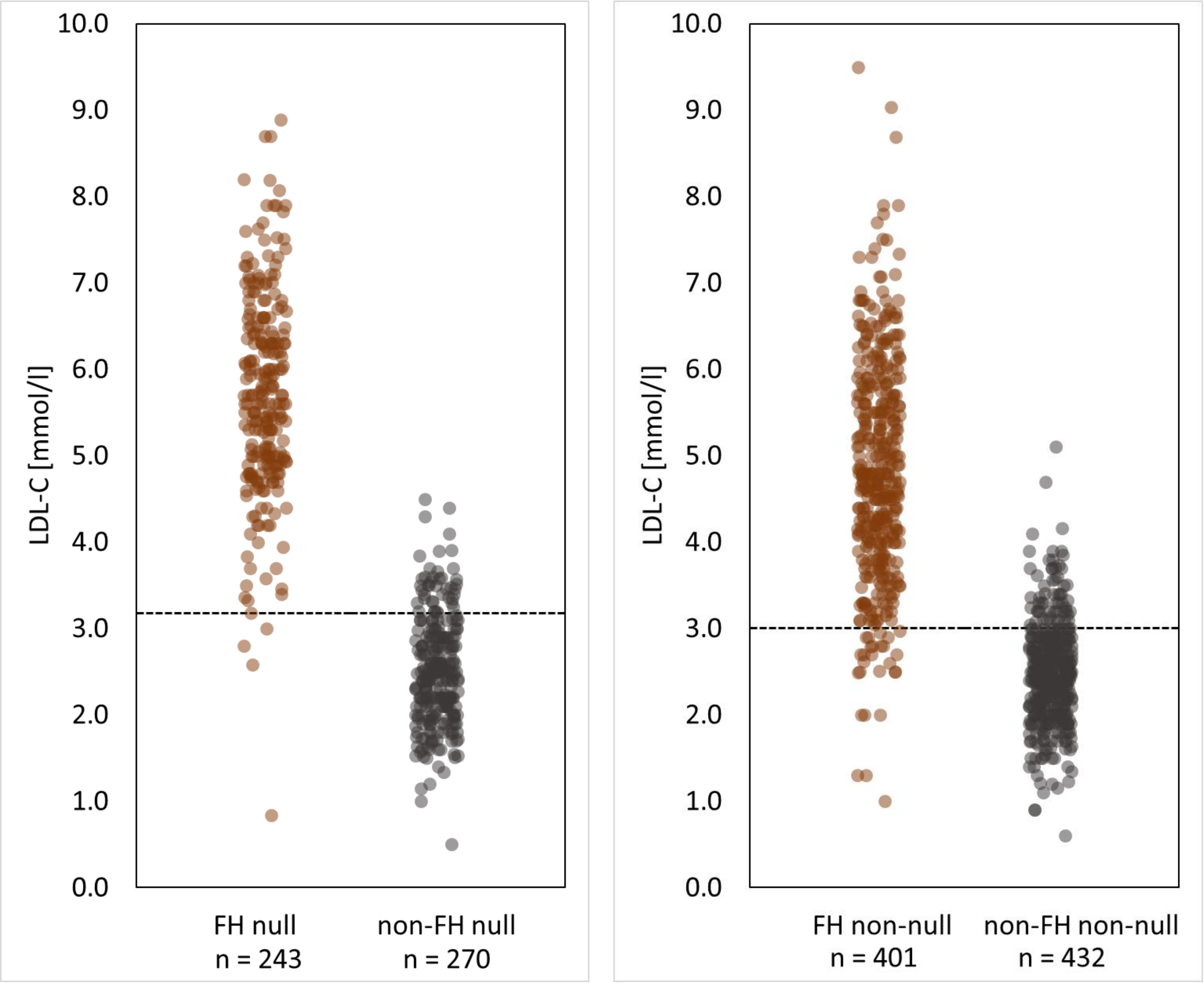

Figure S5. LDL-C in FH and non-FH children 1-12 year old by variant gene.

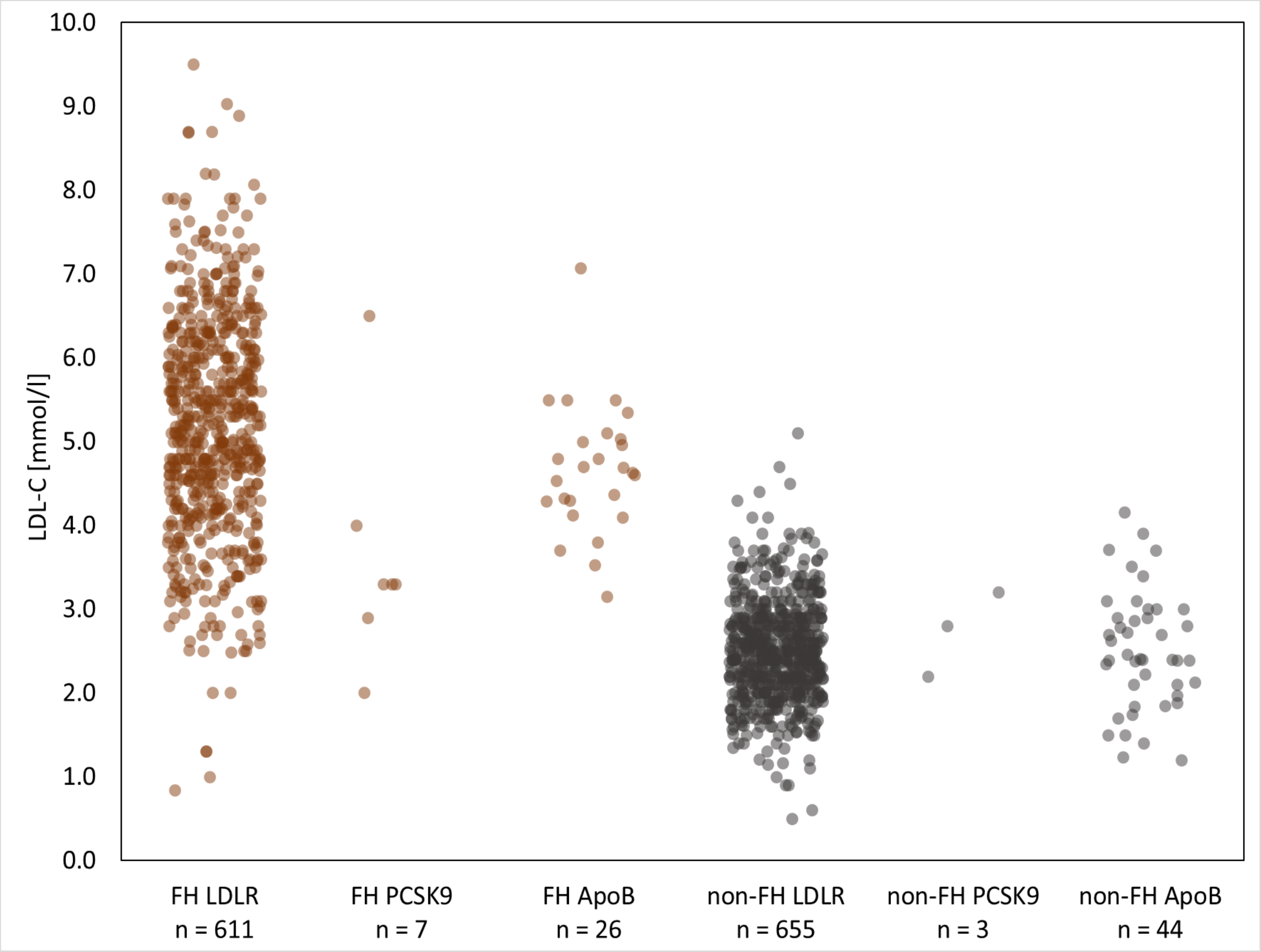

Figure S6. LDL-C in FH and non-FH children 1-12 year old by age.

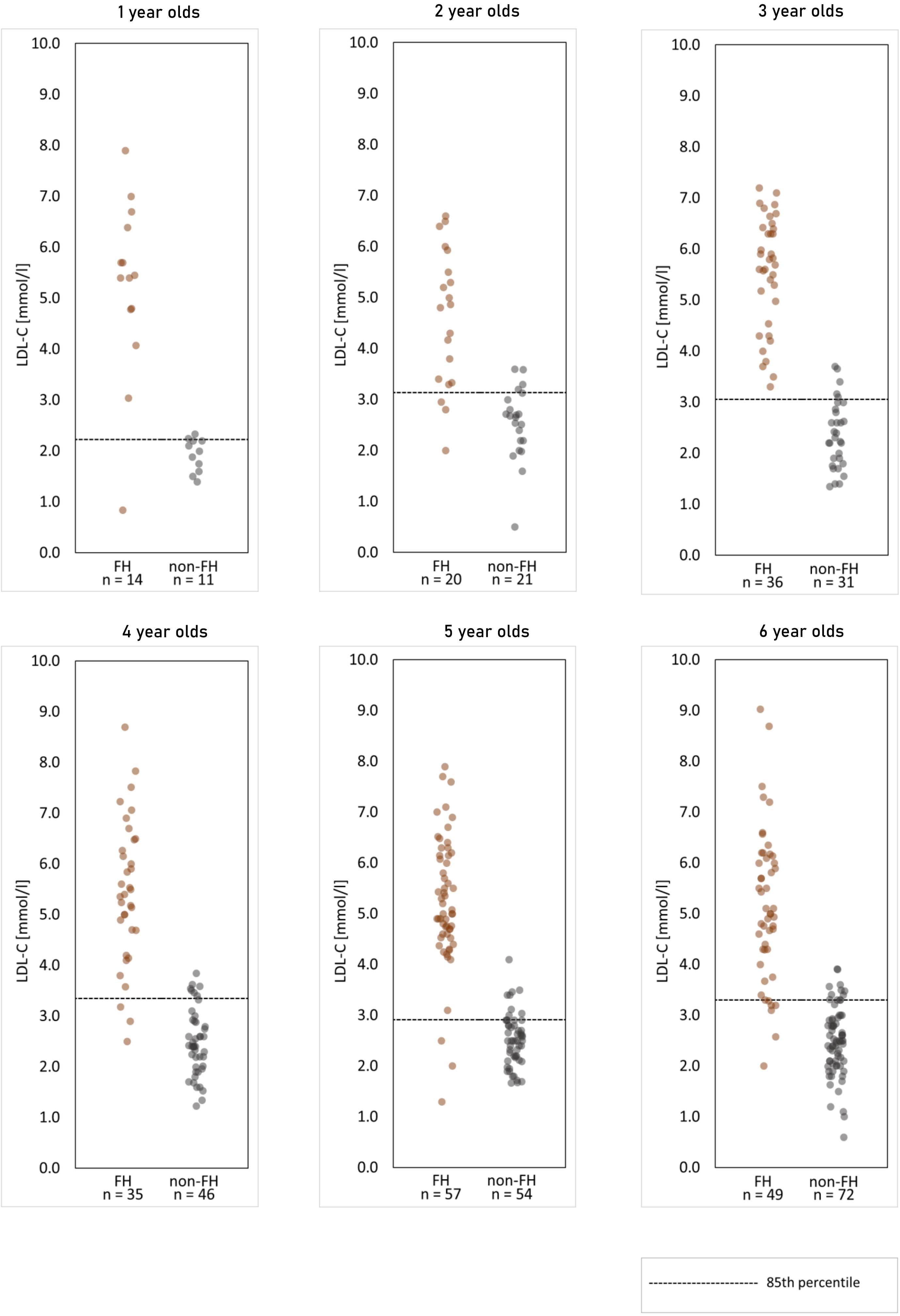

Figure S6 continued.

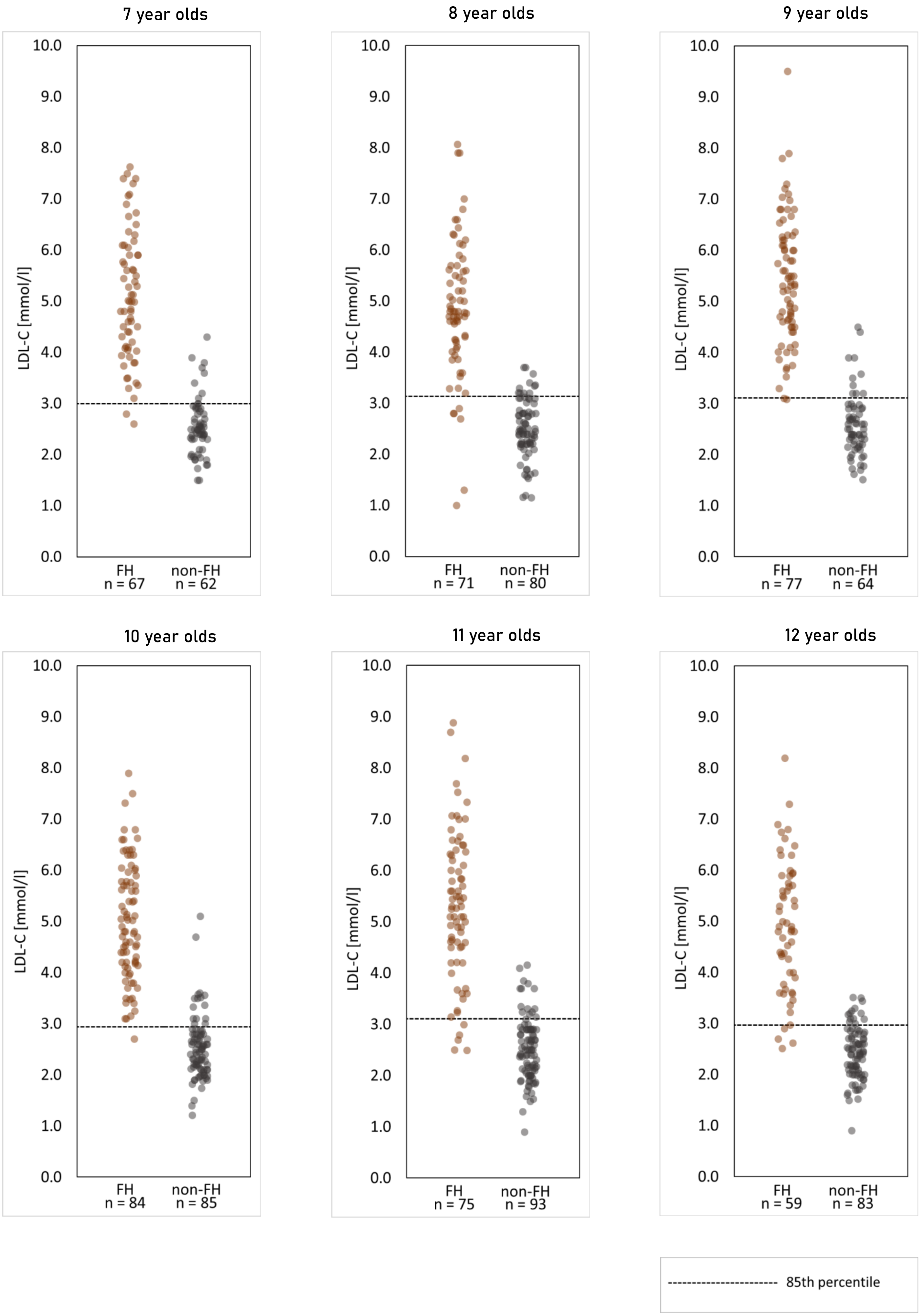

Supplement: ehaf815_Supplementary_Data [file ehaf815_supplementary_data.pdf]
